# Supplementary material for: Superscattering emerging from the physics of bound states in the continuum
Source: Nat Commun. 2023 Aug 4;14:4689. doi: 10.1038/s41467-023-40382-y (PMC10403603; doi:10.1038/s41467-023-40382-y)
Supplement: Supplementary file 1 — Supplementary Information [file 41467_2023_40382_MOESM1_ESM.pdf]

SUPPLEMENTARY INFORMATION
Superscattering Emerging from the Physics of Bound States in the Continuum

Adrià Canós Valero,[1,2] Hadi K. Shamkhi,[2,3] Anton S. Kupriianov,[4] Thomas Weiss,[1] Alexander A. Pavlov,[5] Dmitrii Redka,[6] Vjaceslavs Bobrovs,[7] Yuri S. Kivshar,[8] and Alexander S. Shalin[9,10]

[1] *Institute of Physics, University of Graz, and NAWI Graz, 8010, Graz, Austria*
[2] *ITMO University, St. Petersburg 197101, Russia*
[3] *Institute of Materials Research and Engineering, Agency for Science, Technology and Research, 2 Fusionopolis Way, Innovis #08-03, Singapore 138634, Republic of Singapore*
[4] *College of Physics, Jilin University, Changchun 130012, China*
[5] *Institute of Nanotechnology of Microelectronics of the Russian Academy of Sciences, Moscow 119991, Russia*
[6] *Electrotechnical University LETI, St. Petersburg 197376, Russia*
[7] *Riga Technical University, Institute of Telecommunications, Riga 1048, Latvia*
[8] *Nonlinear Physics Centre, Australian National University, Canberra ACT 2601, Australia*
[9] *Center for Photonics and 2D Materials, Moscow Institute of Physics and Technology, Dolgoprudny 141700, Russia and*
[10] *MSU, Faculty of Physics, Moscow, 119991, Russia*

CONTENTS

S1. QNM Perturbation Theory 2
S2. Connection between Perturbation Theory and TCMT 4
S3. Derivation of Eq.(2) of the main text 5
S4. Derivation of $f(\omega)$ 6
S5. Dependence of the scattering cross section with small ohmic losses 7
S6. Cartesian expressions of the multipole coefficients 7
S7. QNM expansion of the multipole coefficients 8
S8. Experimental Methods 8
S9. Numerical simulations of the lossy ceramic rods 9
S10. Comparison between a super-multipole and conventional superscattering 10
S11. ED resonances in the sphere and the ellipsoid 11
S12. Comparison with previous works on superscattering with non-spherical scatterers 11
References 12

S1. QNM PERTURBATION THEORY

In this section, we introduce a general perturbation scheme to understand the evolution of QNMs of an arbitrary geometry. Later, we present simplified expressions for the coupling between two QNMs. We utilize the latter to analyze the deformation of a sphere to an ellipsoid of revolution in the vicinity of two QNMs radiating as two different multipoles.

In a coupling experiment, one or more parameters of the cavity are varied. In the perturbed system (p), the QNMs are linear combinations of the QNMs of the unperturbed system (0), i.e. $\tilde{\mathbf{E}}_\mu^{(p)} = \sum_\mu C_\mu \tilde{\mathbf{E}}_\mu^{(0)}$. Utilizing first order perturbation theory [1] yields a generalized eigenvalue problem for the 'mixing' coefficients C_μ, (organized in a ket vector for compactness):

$$\tilde{\Omega}^{(0)}|\mathbf{C}\rangle = \tilde{\omega}^{(p)}(\mathcal{U}+\mathcal{V}^{(p)})|\mathbf{C}\rangle, \tag{S.1}$$

$$\mathcal{V}_{ij}^{(p)} = \int_{\partial V} \delta\varepsilon \tilde{\mathbf{E}}_i^{(0+)}(\mathbf{r}) \cdot \delta h(\mathbf{r}) \tilde{\mathbf{E}}_j^{(0-)}(\mathbf{r}) d^2\mathbf{r} = \langle \delta\varepsilon \tilde{\mathbf{E}}_i^{(0)} | \delta h | \tilde{\mathbf{E}}_j^{(0)} \rangle \,, \tag{S.2}$$

where $\tilde{\Omega}^{(0)}$ is a diagonal matrix containing the eigenfrequencies of the unperturbed cavity, and $\mathcal{U}$ is the identity matrix. The $\pm$ in the superscripts indicate the field is evaluated at the outer (+) or inner (-) boundary of the cavity. In what follows, they are omitted for brevity. $\mathcal{V}^{(p)}$ encodes the effect of the perturbation, and each component is determined by overlap integrals of the unperturbed modes over the surface of the cavity ∂V. $\delta h(\mathbf{r})$ is the geometrical deformation, where $\delta h(\mathbf{r})\hat{\mathbf{n}}$ corresponds to a perpendicular shift of the resonator boundary from the undeformed to the deformed surface [1], as illustrated in the insets of Fig. S1(a).

Equations (S.1) and (S.2) are general and can be applied to any open dielectric cavity. This naively seems to imply that arbitrary modal overlaps can occur. However, in canonical geometries, there exist strict symmetry rules that prevent energy exchange between QNMs with different multipolar origin. To illustrate this, consider a sphere of radius R supporting a QNM radiating to the ED channel (a), with $\tilde{\mathbf{E}}_a^{(0)} \propto \tilde{\mathbf{E}}_p(\mathbf{r})$, and a low radiative QNM which only couples to the magnetic quadrupole, labeled b, with $\tilde{\mathbf{E}}_b^{(0)} \propto \tilde{\mathbf{E}}_M(\mathbf{r})$, (the tilde denotes that the multipole fields radiated by each mode must be evaluated at its complex eigenfrequency). The latter effectively corresponds to a quasi-BIC [2]. We first consider the case of a radial deformation [left inset in Fig. S1(a)], which preserves the spherical symmetry. For a bimodal system, the eigenvalue problem in Eq. (S.1) reduces to

$$\begin{pmatrix} \tilde{\omega}_a & 0 \\ 0 & \tilde{\omega}_b \end{pmatrix} \begin{pmatrix} C_a \\ C_b \end{pmatrix} = \tilde{\omega}_{u,d} \begin{pmatrix} 1+V_{aa} & V_{ab} \\ V_{ba} & 1+V_{bb} \end{pmatrix} \begin{pmatrix} C_a \\ C_b \end{pmatrix}. \tag{S.3}$$

The dependence of all V_{ij} elements with the boundary shift has been omitted for clarity. In spherical coordinates, the boundary shift $\delta h(\mathbf{r}) = \delta R$, where δR is a constant determining the increase (or decrease) of the sphere radius. Evaluating the perturbation term V_{ab} then reduces to integrating $\tilde{\mathbf{E}}_p(\mathbf{r}) \cdot \tilde{\mathbf{E}}_M(\mathbf{r})$ over the whole solid angle. Therefore, due to the orthogonality of the multipole fields, $V_{ab} = V_{ba} = 0$ in this first case. Then, the eigenvectors are $|\mathbf{C}_u\rangle = (1,0)^T$ and $|\mathbf{C}_d\rangle = (0,1)^T$, where T denotes transpose. This effectively means that the fields of the perturbed system will be identical to the unperturbed one (not necessarily the eigenfrequencies, which can be shifted since the V_{ii} terms are nonzero). While we have worked on the specific example of ED and MQ modes, the result is general: since multipole fields are orthogonal on the unit sphere, any perturbation that preserves the spherical symmetry *cannot mix* modes matched to different multipole channels.

We now consider the case of interest for this work, namely, the situation when the spherical symmetry is broken. We investigate the transition from a sphere to an ellipsoid of revolution [right inset in Fig. S1(a)]. The boundary shift takes a more complicated form, but it can be shown that, if the ratio between the in-plane and out-of-plane axis of the ellipsoid η is close to unity, to second order in η, $\delta h(\mathbf{r}) = \delta h(\theta) \approx R(1-\eta)\cos^2\theta$, where θ is the polar angle in spherical coordinates. The V_{ab} term is:

$$V_{ab} \propto R^2 \delta\varepsilon (1-\eta) \int_{\partial V} \tilde{\mathbf{E}}_p(\mathbf{r}) \cdot \tilde{\mathbf{E}}_M(\mathbf{r}) \cos^2\theta d\Omega. \tag{S.4}$$

The integral in Eq. (S.4) can be nonzero because (i) the scalar product between two multipole fields of different order does not vanish, and (ii) the function $\cos^2\theta$ prevents the direct application of the orthogonality between the multipole fields in the unit sphere. Since the boundary shift does not depend on the azimuthal angle, the structure retains the rotational symmetry in the x-y plane, so that its point symmetry changes from $O(3)$ to $D_{\infty h}$ (in Schönflies notation). Then, it can be shown through group theoretical arguments that V_{ab} does not vanish, since it couples modes radiating

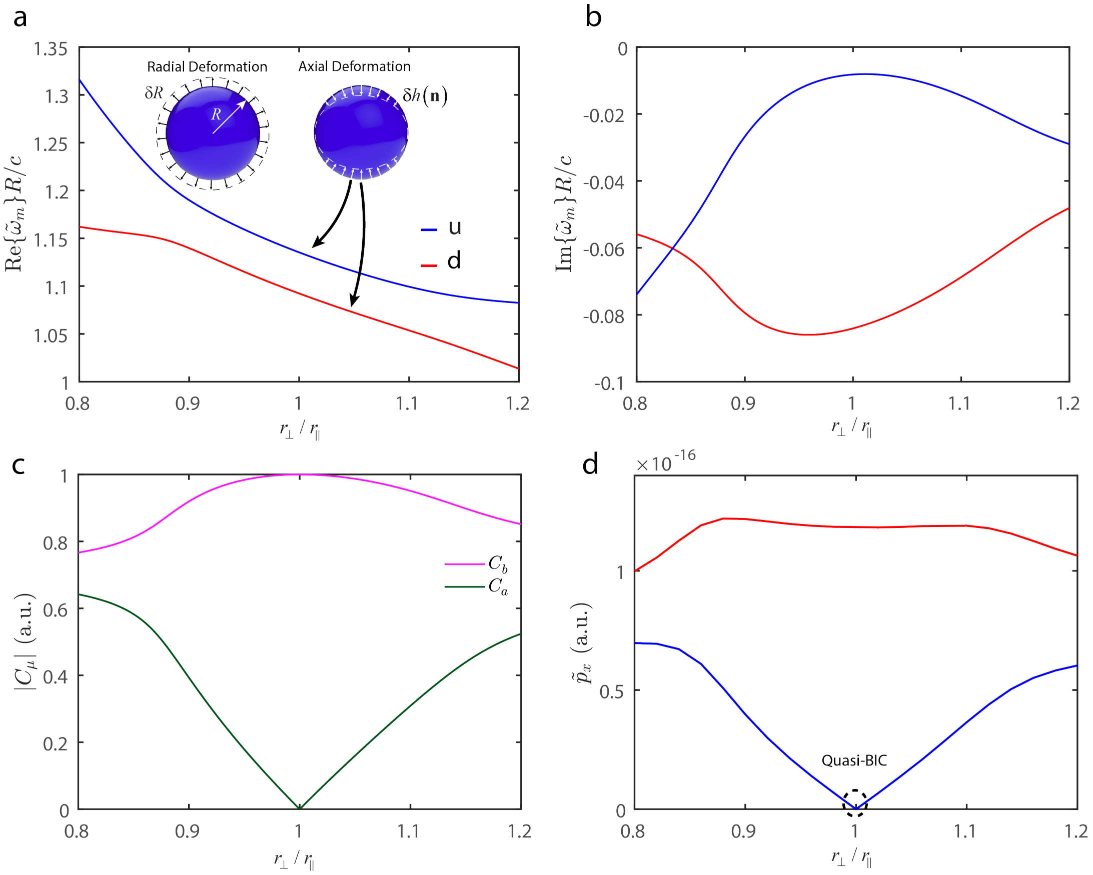

FIG. S1. Predictions of the two-level model given by Eq. (S.3), for the resonant QNMs of a Si sphere with radius 100 nm, as in the first example in the main text . (a) Avoided crossing of the real part of the eigenfrequencies of the hybridized QNMs, u, d as a function of the ratio of out-of-plane to in-plane axis $r_\perp/r_\parallel = 1/\eta$. Inset: the leftmost picture depicts a radial deformation that preserves the spherical symmetry and does not mix the QNMs (corresponding to Ref. **?**). The rightmost one depicts an axial deformation, where the out-of-plane axis of the spheroid is slowly tuned. As explained in the text, the boundary shift $\delta h(\mathbf{n})$ is therefore no longer a constant in spherical coordinates. (b) Imaginary parts of the eigenfrequencies of the two modes as a function of $r_\perp/r_\parallel$. When $r_\perp/r_\parallel = 1$ (a sphere), the imaginary part of QNM u approaches zero, therefore leading to a large $\mathcal{Q}$-factor. (c) Eigenvector components of QNM $\tilde{\mathbf{E}}_u^{(p)}$ expressed in the basis of the original 'pure' multipolar QNMs of the sphere a, b. When $r_\perp/r_\parallel \neq 1$, $V_{ab} \neq 0$, and therefore the mixing angle is nonzero, inducing hybridization between a, b. (d) Intrinsic ED moment of $\tilde{\mathbf{E}}_u^{(p)}$ and $\tilde{\mathbf{E}}_d^{(p)}$. When $r_\perp/r_\parallel = 1$, $\tilde{\mathbf{E}}_u^{(p)} = \tilde{\mathbf{E}}_b^{(0)}$ which radiates only as a magnetic quadrupole (a quasi-BIC). Mode hybridization leads to leakage to the ED channel once $r_\perp/r_\parallel \neq 1$. Conversely, $\tilde{\mathbf{E}}_d^{(p)}$ always retains its dipolar nature, since when $r_\perp/r_\parallel = 1$, $\tilde{\mathbf{E}}_d^{(p)} = \tilde{\mathbf{E}}_a^{(0)}$.

as electric and magnetic multipoles of different order [3], which now belong to the same irreducible representation.

Solving Eq. (S.3) we obtain the perturbed eigenfrequencies $\tilde{\omega}_{u,d} = E \mp \Delta \sec(2\phi)$, where:

$$E = \frac{(1+V_{bb})\tilde{\omega_a} + (1+V_{aa})\tilde{\omega_b}}{2[(1+V_{aa})(1+V_{bb}) - V_{ab}V_{ba}]}, \tag{S.5}$$

$$\Delta = \frac{(1+V_{bb})\tilde{\omega_a} - (1+V_{aa})\tilde{\omega_b}}{2[(1+V_{aa})(1+V_{bb}) - V_{ab}V_{ba}]}, \tag{S.6}$$

$$\tan 2\phi = \frac{V_0}{\Delta}, \tag{S.7}$$

and

$$V_0 = \frac{-\sqrt{\tilde{\omega_a}\tilde{\omega_b}}\sqrt{V_{ab}V_{ba}}}{(1+V_{aa})(1+V_{bb}) - V_{ab}V_{ba}}. \tag{S.8}$$

Similarly, we define the new eigenvectors as:

$$|\mathbf{C}_d\rangle = \begin{pmatrix} -\sqrt{\frac{\tilde{\omega}_b}{\tilde{\omega}_a}}\cos\phi \\ \sin\phi \end{pmatrix}, \tag{S.9}$$

$$|\mathbf{C}_u\rangle = \begin{pmatrix} \sqrt{\frac{\tilde{\omega}_b}{\tilde{\omega}_a}}\sin\phi \\ \cos\phi \end{pmatrix}. \tag{S.10}$$

Equations (S.9) and (S.10) confirm that the new QNMs, once the spherical symmetry is broken, are linear combinations of the QNMs in the sphere. In analogy with a two-level model in quantum physics [4], we call ϕ the *mixing angle*, since it determines the energy exchange between the original QNMs of the sphere. Making the replacement $r = \sqrt{\tilde{\omega}_b/\tilde{\omega}_a}$, Eqs. (S.9) and (S.10) correspond to the hybrid resonances (matched to more than one multipole) discussed in the main text. From the expression of the perturbed eigenfrequencies, we also clearly observe the formation of an *avoided crossing*, which ultimately depends on the coupling strength $V_0 \propto \sqrt{V_{ab}V_{ba}}$.

Notably, if $V_{ab} = 0$, then $\phi = 0$ and there is no mixing, since again we recover $|\mathbf{C}_u\rangle = (1,0)^T$ and $|\mathbf{C}_d\rangle = (0,1)^T$. However, in all other situations there is *hybridization*. This indeed corresponds to the Friedrich-Wintgen mechanism [5].

We are now able to delve into the physics of a realistic nanocavity. In particular, we study the axial deformation of a Si nanosphere with radius 100 nm, discussed in the main text . In Figs. S1(a) and S1(b) we have plotted the real and imaginary parts of the eigenfrequencies recovered with the two-level model in Eq. (S.3). The deformation is parameterized by the ratio between the out-of-plane and in-plane axis of the ellipsoid, $r_\perp/r_\parallel = 1/\eta$.

As expected, we observe an avoided crossing of the real part, as well as a reduced imaginary part for mode u when $r_\perp/r_\parallel = 1$, corresponding to the quasi-BIC condition. In accordance with the two-level picture described in the previous paragraphs, Fig. S1(c) shows how the eigenvector $|\mathbf{C}_u\rangle$, once the spherical symmetry is broken, receives contributions from the two original QNMs. Most importantly, due to the hybridization, mode u can now radiate as an ED. This can be seen in Fig. S1(d), where we have plotted the ED moment of each QNM (evaluated at their eigenfrequency). For QNM u, the ED moment is zero for a sphere, due to the quasi-BIC condition, while QNM d has a large ED moment. Breaking the spherical symmetry inmediately results in coupling between the QNMs: QNM u, due to the hybridization with d, now possesses an ED moment. This mechanism is essential to overcome the single channel limit, as explained in the main text.

S2. CONNECTION BETWEEN PERTURBATION THEORY AND TCMT

In this section we outline the link between QNM perturbation theory and TCMT, and the approximations implied. Based on the link, we obtain suitable analytical forms for the evolution of the TCMT parameters discussed in the main text. A connection can be made by writing the equation for one perturbed QNM, from Eq.(S.3), and comparing it with its counterpart TCMT equation:

$$\tilde{\omega}_a^{(0)} C_a = \tilde{\omega}(1+V_{aa})C_a + \tilde{\omega}V_{ab}C_b, \tag{S.11}$$

$$\tilde{\omega}_a^{(1)} C_a = \tilde{\omega}C_a + i\kappa_{ab}C_b. \tag{S.12}$$

In the previous, we have denoted by $\tilde{\omega}$ the eigenfrequency of the new QNM resulting from the coupling between $|a,b\rangle$. The superscript (1) used in the TCMT equation will become clear in what follows. Dividing the first equation by $1+V_{aa}$ leads to

$$\tilde{\omega}_a^{(1)} C_a = \tilde{\omega}C_a + \frac{\tilde{\omega}}{1+V_{aa}}V_{ab}C_b, \tag{S.13}$$

where $\tilde{\omega}_a^{(1)} = \tilde{\omega}_a^{(0)}/(1+V_{aa})$ represents the evolution that QNM $|a\rangle$ would have in the absence of coupling with $|b\rangle$. Comparing the TCMT equation with the above, it appears that the coupling coefficient can be defined as

$$\kappa_{ab}(\tilde{\omega}) = -i\frac{\tilde{\omega}V_{ab}}{1+V_{aa}}. \tag{S.14}$$

The dependence on $\tilde{\omega}$ can be neglected under the assumption of weak coupling inherent to TCMT. Namely, the effect of $\kappa_{ij}(\tilde{\omega})$ can only be felt in the vicinity of the degeneracy, where $\tilde{\omega}_a^{(1)} \approx \tilde{\omega}_b^{(1)}$. Then, $\tilde{\omega} \approx E_{ab} \pm \delta$, where $E_{ab} = (\tilde{\omega}_a^{(1)} + \tilde{\omega}_b^{(1)})/2$ and δ is a small number, which can be neglected, yielding

$$\kappa_{ij} \approx -i\frac{E_{ab}V_{ij}}{1+V_{ii}}. \tag{S.15}$$

Thus, we can now build the effective Hamiltonian of TCMT as

$$\mathcal{H}_0 = \begin{pmatrix} \tilde{\omega}_a^{(1)} & i\kappa_{ab} \\ i\kappa_{ba} & \tilde{\omega}_b^{(1)} \end{pmatrix}. \tag{S.16}$$

We now impose the second main approximation of TCMT and assume high-Q modes, so that $\kappa_{ab} = -\kappa_{ba}^*$. Further, we assume all coupling takes place in the near field, so that $\kappa_{ab} = \kappa_{ba} = -i\kappa \in i\mathbb{R}$. With this, $\mathcal{H}_0$ takes the simple form given in Eq.(2) in the main text:

$$\mathcal{H}_0 = \begin{pmatrix} \tilde{\omega}_a^{(1)} & \kappa \\ \kappa & \tilde{\omega}_b^{(1)} \end{pmatrix}. \tag{S.17}$$

Finally, to study the Friedrich-Wintgen mechanism, based on the arguments above, we consider the following functional forms:

$$\tilde{\omega}_i^{(1)} = \frac{\tilde{\omega}_i^{(0)}}{1+a_i\zeta}, \tag{S.18}$$

$$\kappa = a_3\zeta, \tag{S.19}$$

where the a_i are constants, representing the surface integrals within the V_{ij}, and ζ is the perturbation, extracted from $\delta h(\mathbf{n})$, assumed to be independent on the spatial coordinates.

S3. DERIVATION OF EQ.(2) OF THE MAIN TEXT

For a given $\mathcal{R}$-matrix, it can be shown that the total scattering cross section stems from the contributions of all scattered multipoles,

$$\sigma = \sum_\tau \sigma_\tau. \tag{S.20}$$

Every σ_τ can be found as [6, 7]:

$$\sigma_\tau = 1/4|\sum_\nu (\mathcal{R}_{\tau\nu} - \delta_{\tau\nu})a_\nu|^2, \tag{S.21}$$

where $\mathcal{R}_{\tau\nu}$ is an element of the reflection matrix $\mathcal{R}(\omega)$ defined in the main text, $\delta_{\tau\nu}$ is the Kronecker delta and $|a_\nu|^2$ is the power carried by the plane wave in the multipole ν [8]. $|a_\nu|^2/I_0 = (2\ell_\nu + 1)\lambda^2/2\pi$, where ℓ_ν is the angular momentum associated with the multipole ν. We consider a hypothetical scenario where channel 1 (for instance the electric dipole), receives contributions from another channel $\tau = 2$. For clarity, we normalize Eq.S.21 by $|a_1|^2 = 3\lambda^2/2\pi = \sigma_{\text{Max}}$ (the limit to the channel cross section in the absence of coupling). The scattering cross section for channel 1 is then given by:

$$\sigma_1(\omega)/\sigma_{\text{Max}} = |f(\omega)|^2, \tag{S.22}$$

$$f(\omega) = \frac{1}{2}(1 - R_{11}(\omega) - R_{12}(\omega)), \tag{S.23}$$

where we have assumed, for simplicity in the toy model, that the power carried by the plane wave in both channels is the same (i.e. $a_1 = a_2$).

S4. DERIVATION OF $f(\omega)$

In this section, we derive expressions for $f(\omega)$ for the two cases considered, (i) single mode approximation, (ii) two mode approximation. We start with (i). Using TCMT, we write the following equations describing the system dynamics:

$$\frac{\partial a}{\partial t} = -i\tilde{\omega}_a a + i\sqrt{2}d_1 s_1^+ + i\sqrt{2}d_2 s_2^+, \tag{S.24}$$

$$s_j^- = s_j^+ + i\sqrt{2}d_j a, \quad (j = 1, 2). \tag{S.25}$$

In Eqs.S.24-S.25, the d_j are the coupling coefficients of the resonant QNM with the incoming waves j. They are related to the radiative losses on each channel as $\gamma_j = d_j^2$, and the total radiative loss is given by $\gamma = \sum_j \gamma_j$. With a time dependence in the form $e^{-i\omega t}$, the $\mathcal{R}$-matrix can be found from

$$\mathcal{R} = I + 2i\frac{\mathcal{M}}{\tilde{\omega}_a - \omega}, \tag{S.26}$$

with $\mathcal{M} = \mathbf{d}\mathbf{d}^T$, and $\mathbf{d}$ is a column vector containing the d_j coefficients. The off-diagonal elements R_{ij} are nonzero due to the term $\mathcal{M}_{12} = d_1 d_2 = \sqrt{\gamma_1\gamma_2}$ of the matrix $\mathcal{M}$. The function $f(\omega)$ in Eq.S.23 can be evaluated as:

$$f(\omega) = i\frac{\gamma_1 + \sqrt{\gamma_1\gamma_2}}{\omega_a - \omega - i(\gamma_1 + \gamma_2)}. \tag{S.27}$$

Next, we consider the system is driven by two resonant QNMs, which corresponds to case (ii). Each one is matched only to a given channel $\tau = 1, 2$. However, the two modes can also interact with each other in the near field, with a coupling constant denoted by κ. Altogether, the TCMT equations can now be formulated as:

$$\frac{\partial a}{\partial t} = -i\tilde{\omega}_a a - i\kappa b + i\sqrt{2}d_1 s_1^+, \tag{S.28}$$

$$\frac{\partial b}{\partial t} = -i\tilde{\omega}_b b - i\kappa a + i\sqrt{2}d_2 s_2^+, \tag{S.29}$$

$$s_1^- = s_1^+ + i\sqrt{2}d_1 a, \tag{S.30}$$

$$s_2^- = s_2^+ + i\sqrt{2}d_2 b. \tag{S.31}$$

Eqs.S.28-S.31 can be written in a more compact form:

$$\frac{\partial \mathbf{A}}{\partial t} = -i\mathcal{H}_0\mathbf{A} + i\sqrt{2}D^T\mathbf{s}^+, \tag{S.32}$$

$$\mathbf{s}^- = \mathbf{s}^+ + i\sqrt{2}D\mathbf{A}, \tag{S.33}$$

where $\mathbf{A}$ is a column vector containing $(|a\rangle, |b\rangle)^T$, $\mathbf{s}^\pm$ is a column vector containing the incoming (or outgoing) amplitudes, $\mathcal{H}_0$ is an *effective Hamiltonian* governing the mode evolution, and D is a diagonal matrix containing the coupling coefficients of the modes to the scattering channels, which reads:

$$D = \begin{pmatrix} d_1 & 0 \\ 0 & d_2 \end{pmatrix}. \tag{S.34}$$

The d_j are connected to the decay rates of each QNM as $\gamma_a = d_1^2$ and $\gamma_b = d_2^2$. If the structure undergoes a suitable geometrical perturbation parameterized by ζ, the QNMs can couple. Within the framework of first order perturbation theory, the effective Hamiltonian $\mathcal{H}_0$ in the basis of the original QNMs reads:

$$\mathcal{H}_0(\zeta) = \begin{pmatrix} \omega_a(\zeta) - i\gamma_a & \kappa(\zeta) \\ \kappa(\zeta) & \omega_b(\zeta) - i\gamma_b \end{pmatrix}, \tag{S.35}$$

the coupling constant $\kappa \in \mathbb{R}$, as discussed in section S2. The new QNMs of $\mathcal{H}_0$ can be expressed in the basis of the unperturbed ones as $|u, d\rangle = (\Delta \pm G)|a\rangle + \kappa|b\rangle$, where $2\Delta = \tilde{\omega}_a - \tilde{\omega}_b$ and $G^2 = E^2 + \kappa^2$, with $2E = \tilde{\omega}_a + \tilde{\omega}_b$. The new fields are necessarily a linear combination of the unperturbed ones. However, the 'hybrid' QNMs no longer diagonalize the $\mathbf{D}$ matrix: each QNM is matched to the two channels, in a proportion dictated by κ. Hereon, it is assumed that

$\gamma_b << \gamma_a$. Moreover, the channels and the modes are in the basis such that radiative losses are minimal for $|b\rangle$ at $\kappa = 0$. Mode $|b\rangle$ is therefore a quasi-BIC.

Therefore, in the presence of coupling between two QNMs matched to different multipoles, the $\mathcal{R}$-matrix is no longer diagonal. Following the same rationale that led to Eq.S.27, we reach the final expression for $f(\omega)$:

$$f(\omega) = \frac{i\gamma_a(\tilde{\omega}_u - \tilde{\omega}_0)}{(\tilde{\omega}_u - \tilde{\omega}_d)(\omega - \tilde{\omega}_u)} - \frac{i\gamma_a(\tilde{\omega}_d - \tilde{\omega}_0)}{(\tilde{\omega}_u - \tilde{\omega}_d)(\omega - \tilde{\omega}_d)}. \tag{S.36}$$

S5. DEPENDENCE OF THE SCATTERING CROSS SECTION WITH SMALL OHMIC LOSSES

As discussed in the main text, the ability to control the $\mathcal{Q}$-factor of the superscattering resonance is a key feature of a super multipole, since it allows to maintain a large scattering cross section even in the presence of ohmic losses. Indeed, large $\mathcal{Q}$-factors come at the disadvantage of stronger dissipation, leading to a steeper drop in the scattering cross section. To demonstrate this, consider a resonance matched to a single multipole $\tau = 1$, having the normalized scattering cross section (at resonance):

$$\sigma = |\frac{\gamma_1}{\gamma_1 + \gamma_0}|^2. \tag{S.37}$$

In the previous, γ_0 denotes the loss rate induced in the mode by absorption. For small absorption, γ_0 can be taken to be proportional to the imaginary part of the refractive index 'δ'. We estimate the rate of change of the scattering cross section with increasing δ as:

$$\frac{d\sigma}{d\delta} \propto -\frac{2}{\gamma_1} = \frac{4\mathcal{Q}}{\omega_1}, \tag{S.38}$$

where $\mathcal{Q} = \omega_1/2\gamma_1$ is the original $\mathcal{Q}$-factor in the absence of ohmic losses, and ω_1 is the resonance frequency. A similar expression can be derived in the presence of leakage to multiple radiation channels.

S6. CARTESIAN EXPRESSIONS OF THE MULTIPOLE COEFFICIENTS

It is sometimes more convenient to express each scattering channel τ in the Cartesian basis [9]. We emphasize that each multipole moment (whether expressed in the spherical or the Cartesian basis) constitutes a scattering channel, and the contribution to the cross section in a sphere is limited to the usual bound. Recently, elegant formulae for the multipole moments in the Cartesian basis in terms of the source currents have been proposed [9]. We provide them here for consistency, with a modification of the prefactors to account for power normalization:

$$\mathbf{p} = C^p \int \mathbf{P} j_0(k\rho) d^3\mathbf{r} + \frac{k^2 C^p}{10} \int \left\{ [\mathbf{r}\cdot\mathbf{P}]\mathbf{r} - \frac{1}{3}\rho^2 \mathbf{P} \right\} \frac{15 j_2(k\rho)}{(k\rho)^2} d^3\mathbf{r}, \tag{S.39}$$

$$\mathbf{m} = -\frac{3i\omega C^m}{2} \int (\mathbf{r}\times\mathbf{P}) \frac{j_1(k\rho)}{k\rho} d^3\mathbf{r}, \tag{S.40}$$

$$M = \frac{\omega C^M}{3i} \int \{[\mathbf{r}\times\mathbf{P}]\otimes\mathbf{r} + \mathbf{r}\otimes[\mathbf{r}\times\mathbf{P}]\} \frac{15 j_2(k\rho)}{(k\rho)^2} d^3\mathbf{r}, \tag{S.41}$$

$$\begin{aligned} Q = C^Q \int \{3(\mathbf{r}\otimes\mathbf{P} + \mathbf{P}\otimes\mathbf{r})\} \times \frac{3 j_1(k\rho)}{k\rho} d^3\mathbf{r} + \\ 6k^2 C^Q \int \left\{ 5\mathbf{r}\otimes\mathbf{r}[\mathbf{r}\cdot\mathbf{P}] - (\mathbf{r}\otimes\mathbf{P} + \mathbf{P}\otimes\mathbf{r})\rho^2 \right\} \frac{j_3(k\rho)}{(k\rho)^3} d^3\mathbf{r}. \end{aligned} \tag{S.42}$$

The prefactors in Eqs. (S.39)-(S.42) are:

$$C^p = \frac{k^2}{2\varepsilon_0\sqrt{3\pi Z_0}}, \tag{S.43}$$

$$C^m = \frac{k^2}{2\sqrt{3\pi\varepsilon_0 c}}, \tag{S.44}$$

$$C^Q = \frac{k^3}{4\varepsilon_0\sqrt{10\pi Z_0}}, \tag{S.45}$$

$$C^M = \frac{k^3}{4\sqrt{10\pi\varepsilon_0 c}}. \tag{S.46}$$

$$\tag{S.47}$$

The integrals in Eqs. (S.39)-(S.42) are performed over the resonator volume. The polarization currents $\mathbf{P}$ are defined as $\mathbf{P} = \delta\varepsilon(\mathbf{r})\mathbf{E}(\mathbf{r})$, where $\delta\varepsilon(\mathbf{r}) = \varepsilon(\mathbf{r}) - \varepsilon_0$ is the permittivity contrast between the resonator and free space, and $\mathbf{E}(\mathbf{r})$ is the total field *within* the resonator. For clarity, the spatial dependence of $\mathbf{P}$ has been omitted everywhere.

S7. QNM EXPANSION OF THE MULTIPOLE COEFFICIENTS

QNMs form a complete basis for the total field inside the resonator [10]. Consequently, we can write:

$$\mathbf{E}(\mathbf{r};\omega)\theta(\mathbf{r}) = \sum_{\mu} \alpha_\mu(\omega)\tilde{\mathbf{E}}_\mu(\mathbf{r}), \tag{S.48}$$

where $\theta(\mathbf{r})$ is a function that equals one within the volume of the resonator V, and zero outside. There are several equivalent forms for the expansion coefficients $\alpha_\mu(\omega)$ [10, 11]. Assuming a non-dispersive material, normalizing the QNMs with the Sauvan norm [12], and making use of reciprocity arguments, the $\alpha_\mu(\omega)$ are given by [10]:

$$\alpha_\mu(\omega) = -\frac{\omega}{\omega - \tilde{\omega}_\mu}\int_V \delta\varepsilon \mathbf{E}_b(\mathbf{r};\omega)\cdot\tilde{\mathbf{E}}_\mu(\mathbf{r})d^3\mathbf{r}. \tag{S.49}$$

The background field is, in our case, a normally incident plane wave, e.g. $\mathbf{E}_b(\mathbf{r};\omega) = E_0 e^{ikz}\mathbf{e}_x$. We can now define the QNM contributions to the total polarization current as $\tilde{\mathbf{P}}_\mu = \delta\varepsilon\tilde{\mathbf{E}}_\mu$, so that $\mathbf{P} = \sum_\mu \alpha_\mu \tilde{\mathbf{P}}_\mu$. Substituting this expression into the multipole moments in Eqs. (S.39)-(S.42) yields a rigorous QNM expansion of the multipole moments at real or complex frequencies.

S8. EXPERIMENTAL METHODS

A Taizhou Wangling TP-series microwave ceramic composite is used as a dielectric material for resonators (disks) fabrication. According to the manufacturer data sheet, relative permittivity of this ceramic is 22 ± 1 and loss tangent is $\tan\delta \approx 1 \cdot 10^{-3}$ at 10 GHz. A set of disks is fabricated from the ceramic plates with the use of a precise milling machine. The radius of disks is 4 mm. The sample to be measured is composed of several disks to obtain several resonators with thicknesses of 6.0, 6.9, and 10.0 mm. The sample is mounted on a support made of ROHACELL® 71 HF plate, whose relative permittivity is 1.01 ± 0.05.

To measure the total extinction cross section, the sample is placed in an echoic chamber and illuminated by a normally incident linearly-polarized waves radiated and received by a pair of HengDa Microwave HD-10180DRA10 horn antennas. A photograph of the experimental setup is shown in Fig. S2(a). The antennas operating range is 1–18 GHz. The distance between each horn antenna and the sample is fixed at 2.0 m. The antennas are connected to the ports of Rhode & Schwarz ZVA50 Vector Network Analyzer (VNA) by 50-Ω coaxial cables. Using VNA the $S21$-parameter (transmission coefficient) is detected and analyzed by a special computer software. Forward scattering is obtained from the measured transmission coefficient. The total extinction cross section is extracted from the measured complex magnitude of the forward-scattered signal by means of the optical theorem [13]. A stainless steel sphere with the radius 40.00 ± 0.04 mm and the corresponding analytical Mie solution are used for the signal calibration. To reduce unwanted reverberations between antennas the time gating technique is used. Then a standard

filtering procedure is applied to the raw data to remove the background noise.

A photograph of the measurement setup for mapping the electric near-field is presented in Fig. S2(b). The transmitting antenna generates a linearly polarized quasi-plane-wave whose polarization is orthogonal to the symmetry axis (the z axis) of the disk resonator. The receiving antenna is substituted by an electrically small probe. This probe is a Hertz dipole which is oriented along the polarization direction of the incident wave. The probe detects the dominant component (E_x) of the scattered electric near-field. A LINBOU near-field imaging system is used for the near-field mapping. The scan area around the sample has dimensions 50×50 mm^2 in the x-z plane. In the measurements, the probe automatically moves in the scan area with a 1-mm step along two orthogonal directions. At each probe position, both the amplitude and the phase of the scattered electric field are measured. The exception is the area near the sample. This area is experimentally inaccessible because of the technical limitation on the distance between the moving probe and the vertically standing sample. In the next section we provide further details on the numerical simulations of the ceramic rods utilized in the experiment.

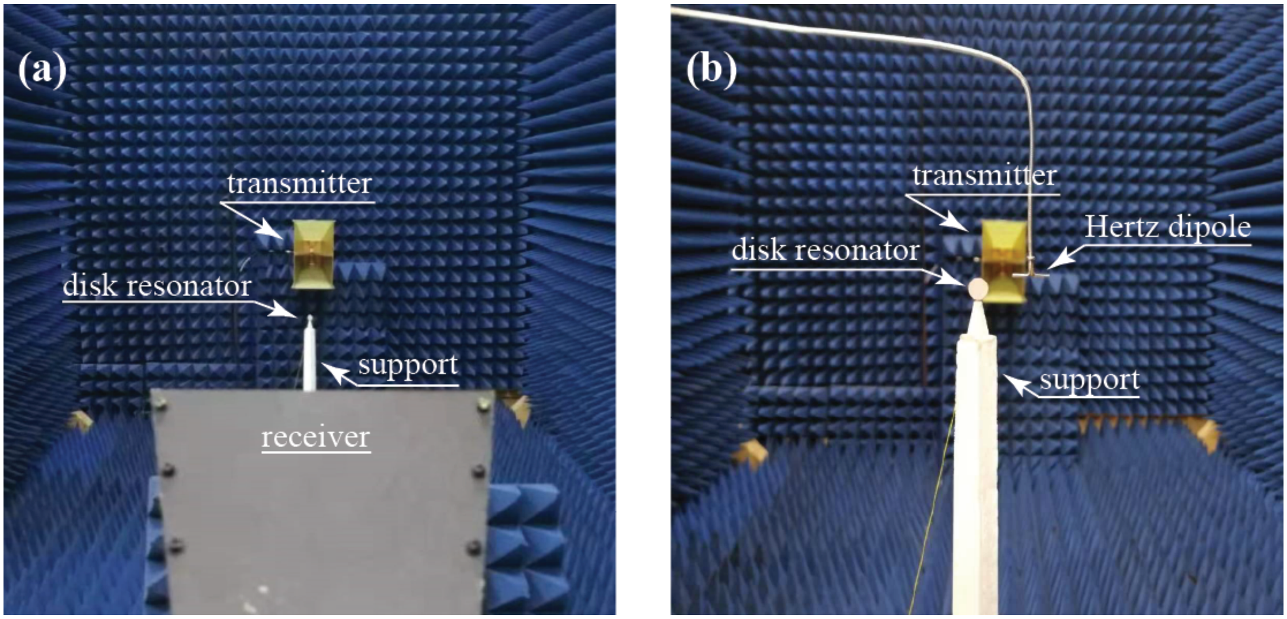

FIG. S2. Experimental setups for (a) measurement of the extinction cross section and (b) mapping of the electric near-field pattern.

S9. NUMERICAL SIMULATIONS OF THE LOSSY CERAMIC RODS

Figure S3 presents the results of our numerical simulations of the scattering cross section for the lossy ceramic rods with parameters corresponding to those of the experimental samples. Here, once again, we obtain the contributions of each multipole for different aspect ratios. We indeed observe the formation of super-ED modes on the upper [Fig. S3(a)] and lower [Fig. S3(c)] branches depending on the aspect ratio. Near the quasi-BIC condition [Fig. S3(b)], a sharp MQ resonance appears in the upper branch that is a signature of the decoupling from the ED channel.

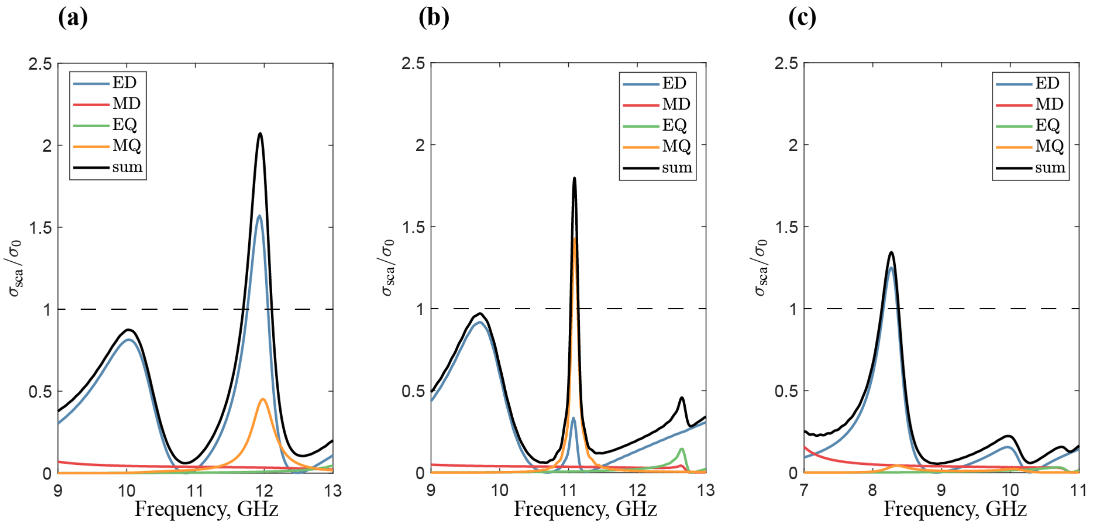

FIG. S3. Multipolar decomposition of the normalized total scattering cross section of the lossy ceramic cylinders presented in the main text (Fig. 4), with $\varepsilon = 22$ and loss tangent 0.001. The cylinder is illuminated with a normally incident, x-polarized plane wave. The aspect ratios are: (a) $\Delta h/r = 0.25$, (b) $\Delta h/r = 0.475$, and (c) $\Delta h/r = 1.25$.

S10. COMPARISON BETWEEN A SUPER-MULTIPOLE AND CONVENTIONAL SUPERSCATTERING

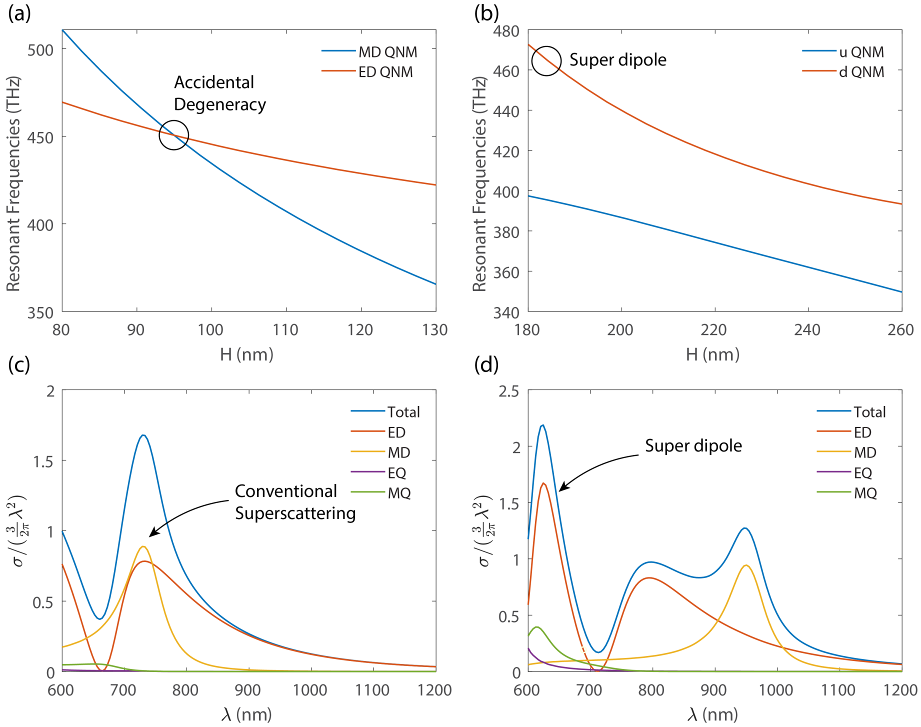

FIG. S4. Comparison between the conventional superscattering effect and a super dipole resonance. The numerical results are obtained for a series of Si nanocylinders of r=126 nm and varying height H. (a) Resonant frequencies of the MD and ED QNMs in the vicinity of an accidental degeneracy. (b) Avoided crossing of the two hybrid QNMs studied in the main text. (c) Multipole decomposition for H=95 nm, corresponding to the maximal overlap between the ED and MD QNMs. The multipoles do not exceed the single channel limit, but their sum does. (d) Super dipole resonance, also indicated by the black circle in panel (b). Here, the electric dipole can exceed the single channel limit, and there is no need for crossing of the resonant frequencies.

S11. ED RESONANCES IN THE SPHERE AND THE ELLIPSOID

In this section we compare the scattering cross section of the first ED resonances in a Si sphere ($n = 4$) vs. an ellipsoid, to further demonstrate the correctness of the single-channel limit in the main text (Fig.S5). For the sphere, all the resonances are always bounded to the single channel limit. However, the optimized ellipsoid can greatly surpass this limit. Moreover, the maximum enhancement can be clearly seen to occur at the super ED resonance described in the main text.

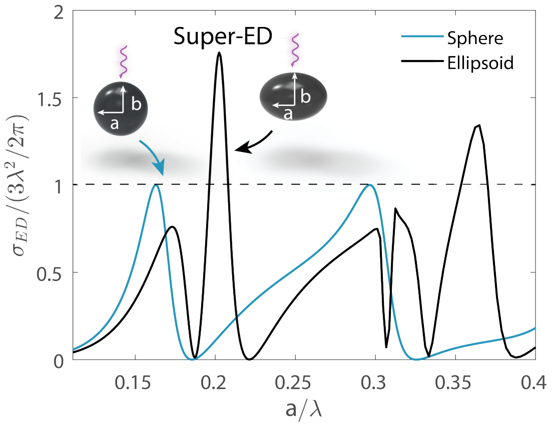

FIG. S5. Lowest order ED resonances of a Si sphere and a Si ellipsoid (refractive index fixed at $n = 4$), with aspect ratio $b/a = 0.8$ vs. a/λ. b, a are the semiaxis of the ellipsoid (or the radius of the sphere). a is kept fixed at 100 nm. For the sphere, all the resonances are bounded to the single channel limit. However, the optimized ellipsoid can greatly surpass this limit. Moreover, the maximum enhancement can be clearly seen to occur at the super ED resonance described in the main text. Only the ED scattering cross section is plotted.

S12. COMPARISON WITH PREVIOUS WORKS ON SUPERSCATTERING WITH NON-SPHERICAL SCATTERERS

In this section we study in more detail previous works [14, 15] investigating non-spherical scatterers with enhanced scattering, and demonstrate that they do not display super multipoles. First, we consider the cylindrical resonator investigated in Ref.[14]. In the latter, the authors presented a Ag nanocylinder embedded in a polymer with refractive index 1.4, and claimed to reach the superscattering regime. We note that all the cross sections in their work are normalized to arbitrary units, making it difficult to determine the scattering enhancement. In consequence, we have reproduced their simulations with the help of COMSOL multiphysics. Fig.S6 shows our calculations of the scattering cross section using the same parameters (details are given in the caption). The calculated scattering cross section is only slightly above the single-channel limit (approximately 1.2 times). We attribute the small efficiency to the presence of Ohmic losses from Ag. Most importantly, the mild enhancement is due to the spectral overlap of the ED and EQ resonances, which are associated to two distinct eigenmodes of the cylinder [Fig. S6(b)]. Each multipole, by itself, does not exceed the single-channel limit. Hence, despite investigating a similar geometry, the authors did not observe the same effect that we are describing in our work, where a *single multipole* exceeds the limit.

Next, we consider the structures investigated in Ref.[15]. The authors considered Ag ellipsoids of extreme subwavelength sizes, in the order of 6% the wavelength, [as depicted in the inset of Figure S6(c)]. We have calculated the scattering cross section of one of such ellipsoids near their localized plasmon resonance, [Figure S6(c)]. The scattering cross section is 1000 times below the single channel limit. Therefore, these ellipsoids are very weak scatterers. Moreover, Figure S6(d) compares the magnitudes of the scattering and absorption efficiencies. Absorption is almost 20 times larger. To understand these results, we should clarify that the authors in Ref.[15] were not interested in maximizing the scattering cross section of a single particle; their figure of merit was the extinction per unit volume. In addition, as we have now verified with full-wave simulations, the response of such particles will always be strongly dominated by absorption, in contrast to our dielectric nanoparticles.

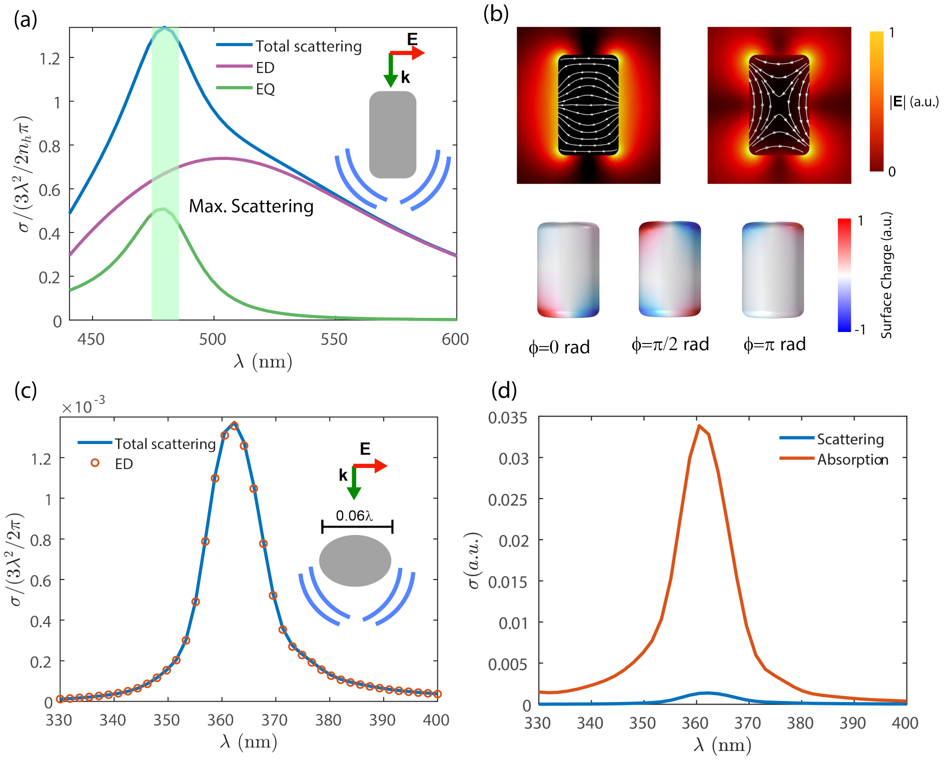

FIG. S6. Comparison with earlier works that demonstrated enhanced scattering with non-spherical shapes in/ near the visible range. (a)-(b) cylindrical plasmonic nanocavity studied in Ref. [14]. Following the latter, the nanoparticle is described by the Drude model $\varepsilon(\omega) = \varepsilon_\infty - \frac{\omega_p^2}{\omega(\omega+i\gamma_c)}$, with the following parameters: $\varepsilon_\infty = 4$, $\omega_p = 1.336 \cdot 10^{16}$ rad/s, $\gamma_c = 1.108 \cdot 10^{14}$ rad/s . The scatterer is embedded in a host environment with refractive index $n_h = 1.4$, hence the single channel limit is given by $\sigma_{\text{Max}} = \frac{3}{3\pi n_h}\lambda^2$. The diameter of the cylinder is 80 nm, and the height is 120 nm. All corners have been rounded to avoid unphysical field singularities. (a): Multipole decomposition of the scattering cross section. Inset: scheme depicting the illumination setup. The shaded green area indicates the spectral region with maximum scattering enhancement. The enhanced scattering occurs due to the overlap of the ED and EQ contributions, following the conventional mechanism of Fan et al [6, 8]. Note that none of the two multipoles exceeds the single channel limit, and the total cross section is only slightly above it. (b) Upper panels: field distributions of the two involved QNMs. The rightmost panel is the ED QNM, and the leftmost is the EQ QNM. Lower panels: Evolution of the surface charge during half an oscillation period at the wavelength with maximum scattering enhancement. (c)-(d) Ag ellipsoids investigated in Ref.[15]. (c) Multipole decomposition of the scattering cross section of an ellipsoid composed of Ag (material data obtained from Johnson and Christy [16]), with semi-major axis 10 nm. The scattering cross section is entirely dominated by the ED, and shows values almost 1000 times below the single channel limit. Inset: scheme of the illumination setup and the scatterer under consideration. (d) Comparison between scattering and absorption efficiencies (in arbitrary units). The absorption cross section is almost 20 times larger than the scattering cross section.

REFERENCES

[1] Wei Yan, Philippe Lalanne, and Min Qiu. "Shape deformation of nanoresonator: A quasinormal-mode perturbation theory". In: *Phys. Rev. Lett.* 125.1 (2020), p. 013901.

[2] Andrey A Bogdanov et al. "Bound states in the continuum and Fano resonances in the strong mode coupling regime". In: *Adv. Photonics* 1.1 (2019), p. 016001.

[3] Sergey Gladyshev, Kristina Frizyuk, and Andrey Bogdanov. "Symmetry analysis and multipole classification of eigenmodes in electromagnetic resonators for engineering their optical properties". In: *Phys. Rev. B* 102.7 (2020), p. 075103.

[4] R. P. Feynman. *Feynman Lectures on Physics. Quantum Mechanics.* Ed. by R. P. Feynman, R. B. Leighton, and M. Sands. Vol. 3. Massachusetts: Addison-Wesley, 1964.

[5] H. Friedrich and D. Wintgen. "Interfering resonances and bound states in the continuum". In: *Phys. Rev. A* 32 (6 Dec. 1985), pp. 3231–3242. DOI: 10.1103/PhysRevA.32.3231.

[6] Zhichao Ruan and Shanhui Fan. "Superscattering of light from subwavelength nanostructures". In: *Phys. Rev. Lett.* 105.1 (2010), p. 013901.

[7] Zhichao Ruan and Shanhui Fan. "Temporal coupled-mode theory for Fano resonance in light scattering by a single obstacle". In: *The journal of physical chemistry C* 114.16 (2010), pp. 7324–7329.

[8] Zhichao Ruan and Shanhui Fan. "Design of subwavelength superscattering nanospheres". In: *Appl. Phys. Lett.* 98.4 (2011), p. 043101.

[9] Rasoul Alaee, Carsten Rockstuhl, and Ivan Fernandez-Corbaton. "An electromagnetic multipole expansion beyond the long-wavelength approximation". In: *Opt. Commun.* 407 (2018), pp. 17–21.

[10] Philippe Lalanne et al. "Light interaction with photonic and plasmonic resonances". In: *Laser Photonics Rev.* 12.5 (2018), p. 1700113.

[11] Philip Trøst Kristensen et al. "Modeling electromagnetic resonators using quasinormal modes". In: *Adv. Opt. Photon.* 12.3 (2020), pp. 612–708.

[12] Christophe Sauvan et al. "Theory of the spontaneous optical emission of nanosize photonic and plasmon resonators". In: *Phys. Rev. Lett.* 110.23 (2013), p. 237401.

[13] R. G. Newton. "Optical theorem and beyond". In: *Am. J. Phys.* 44.7 (1976), pp. 639–642. DOI: 10.1119/1.10324.

[14] Weiwei Wan et al. "From Fano-like interference to superscattering with a single metallic nanodisk". In: *Nanoscale* 6.15 (2014), pp. 9093–9102.

[15] Owen D Miller et al. "Fundamental limits to extinction by metallic nanoparticles". In: *Physical review letters* 112.12 (2014), p. 123903.

[16] Peter B Johnson and R-WJPrB Christy. "Optical constants of the noble metals". In: *Physical review B* 6.12 (1972), p. 4370.
